# Spectroscopic Investigation of the Kinetic Mechanism Involved in the Association of Potyviral VPg with the Host Plant Translation Initiation Factor eIF4E

**DOI:** 10.3390/ijms21165618

**Published:** 2020-08-05

**Authors:** Jocelyne Walter, Amandine Barra, Justine Charon, Geneviève Tavert-Roudet, Thierry Michon

**Affiliations:** 1 INRAE, Biologie du Fruit et Pathologie, University of Bordeaux, UMR 1332, F-33140 Villenave d’Ornon, France; jocelyne.walter@inrae.fr (J.W.); amandine.barra@inrae.fr (A.B.); genevieve.roudet@inrae.fr (G.T.-R.); 2 Faculty of Sciences, University of Sydney, Charles Perkins Center D17, Camperdown Campus, Sydney, NSW 2006, Australia; justine.charon@sydney.edu.au

**Keywords:** intrinsically disordered protein, eIF4E, VPg, potyvirus, induced folding, protein–protein interaction, pre-steady state kinetics

## Abstract

The infectious cycle of potyviruses requires the formation of a complex between the viral genome-linked protein VPg and the host eukaryotic translation initiation factor 4E, eIF4E. Mutations associated with plant resistance to potyviruses were previously mapped at the eIF4E surface, while on the virus side, mutations leading to plant resistance breaking were identified within the VPg. In the present study, fluorescence spectroscopy was used to probe the contribution of the VPg intrinsically disordered region bearing amino acids determinant of the resistance breaking, to the VPg–eIF4E binding mechanism. Synthetic peptides encompassing the VPg_88–120_ central region were found to tightly bind to eIF4E. Fluorescence energy transfer experiments show that, upon binding to eIF4E, the N and C termini of the VPg_88–111_ fragment move closer to one another, at a distance compatible with a α-helix folding. When the VPg_112–120_ region, which contains amino acids associated with resistance breakdown, is appended to VPg_88–111_, the complex formation with eIF4E switches from a single-step to a two-step kinetic model. This study revisits a recent investigation of the VPg–eIF4E complex by specifying the contribution of the VPg central helix and its appended disordered region to VPg association with eIF4E.

## 1. Introduction

The genus *Potyvirus* represents one of the largest and most economically damaging genus of plant-infecting viruses [1]. Because of their wide host range and wide spread geographical occurrence, potyviruses are very difficult to contain and cause dramatic losses in cultural crops worldwide. Consequently, the molecular biology of potyviruses and the function of their various proteins are thoroughly studied as a necessary step for developing new resistance strategies in open-field farming [2,3]. These viruses possess a single-stranded, polyadenylated, positive-sense genomic RNA, that is covalently linked at its 5′ end to a viral protein, the viral genome-linked protein VPg [4,5]. The potyviral VPg is a virulence factor which has been shown to interact with several viral and host factors and is assumed to be a multifunctional protein involved in essential steps of the virus infectious cycle, such as translation, replication and movement [6,7]. VPg recruits the eukaryotic translation initiation factor 4E (eIF4E), or its isoform eIF(iso)4E. This interaction is crucial for infection [8,9,10]. The general structural similarity between the potyvirus genomic RNA and eukaryotic mRNAs suggests that VPg may serve as a cap-like structure, allowing the virus to recruit the cell translation machinery. However, it was reported that potyviral genome translation initiation may also occur at the 5′ UTR through an internal ribosome entry site (IRES) [11]. Furthermore, the VPg-mediated enhancement of viral genome expression was observed as concomitant with a cellular mRNA expression shut down. It was suggested that VPg could inhibit endogenous mRNA translation by complexing available eIF4E [12]. The phosphorylation of the eIF4E isoform enhances eIFiso4E–VPg binding and mRNA translation in vitro [13]. Furthermore, we observed that the in planta recruitment by mature lettuce mosaic virus (LMV) viral particles of a unique eIF4E molecule linked to the VPg increases LMV infectious ability in vivo [14]. Some pepper cultivars bear a recessive resistance gene to potato virus Y (PVY), encoding eIF4E. The eIF4E mutations associated with this resistance were correlated in some cases with an impairment of the eIF4E interaction with VPg. Correspondingly, mutations in the central region of VPg (residues 80–125) are associated with resistance breakdown [15,16,17]. In the LMV VPg, this central region interacts both with eIF4E and the virus helper component HcPro [18]. VPg, either alone or in combination with eIF4E, has the hydrodynamic behavior of a pre-molten globule, a class of proteins containing both elements of secondary structure and intrinsically disordered regions [19,20]. This was confirmed in the recently solved 3D structure of the VPg from PVY. A model of its complex with human eIF4E was proposed [21]. In this complex, the VPg region residues 111 to 121 within the VPg central region are indeed involved in the interaction with eIF4E. However, the helix spanning from residue 93 to 110 does not seem to be part of the interacting surface. Surprisingly, we found that a synthetic peptide encompassing the 88–111 region of the LMV VPg strongly binds to the lettuce eIF4E. In this study, we used fluorescence microscopy to dissect the kinetic mechanism by which this region binds to eIF4E.

## 2. Results and Discussions

### 2.1. Investigation of the eIF4E–VPg Interaction Domains

Lettuce eIF4E mutations associated with resistance to LMV are not correlated with modulations of the VPg/eIF4E strength of interaction. A correlation between resistance to potyviruses and a defect in the interaction between VPg and eIF4E was reported for PVY-Capsicum annuum and TuMV-Brassica perviridis pathosystems [8,9]. As for other potyviruses, the VPg of LMV interacts with the eIF4E from lettuce in vitro [18,22]. Thermodynamic dissociation constants between the VPg from the non-resistance-breaking isolate LMV0 and two forms of eIF4E carrying mutations conferring resistance to LMV [23], namely eIF4E^1^ (∆ (108–110)H defined as residues 108-110 substituted by a single histidine and A186S) and eIF4E^2^ (A70P), were determined by steady state fluorescence measurements (Table 1). The VPg from a non-resistance-breaking LMV isolate (LMV0) binds to eIF4E from susceptible (eIF4E^0^) and resistant (eIF4E^1^ and eIF4E^2^) lettuce cultivars with similar affinities (Table 1). These results were confirmed by ELISA experiments in which binary interactions between eIF4E^1^ or eIF4E^2^ and VPgs from LMV0 or resistance-breaking (LMVE and LMV-AF199) isolates were tested (Figure S1 in Supplementary Materials). Hence, according to our data, no obvious direct correlation can be made between infectivity and the VPg–lettuce eIF4E interaction in vitro. VPg and m7GTP, the cap analogue, compete for their binding to eIF4E. However, no significant differences in this competition could be observed when measurements were made with eIF4E^1^ and eIF4E^2^ originating from lettuce resistance cultivars. To sum up, there is no obvious correlation between apparent thermodynamic equilibrium constants of VPg–eIF4E association and the resistance function carried by eIF4E^1^ and eIF4E^2^, as shown in Table 1.

#### 2.1.1. The eIF4E C-ter Domain Is Involved in the Interaction with VPg

**Figure 1:**
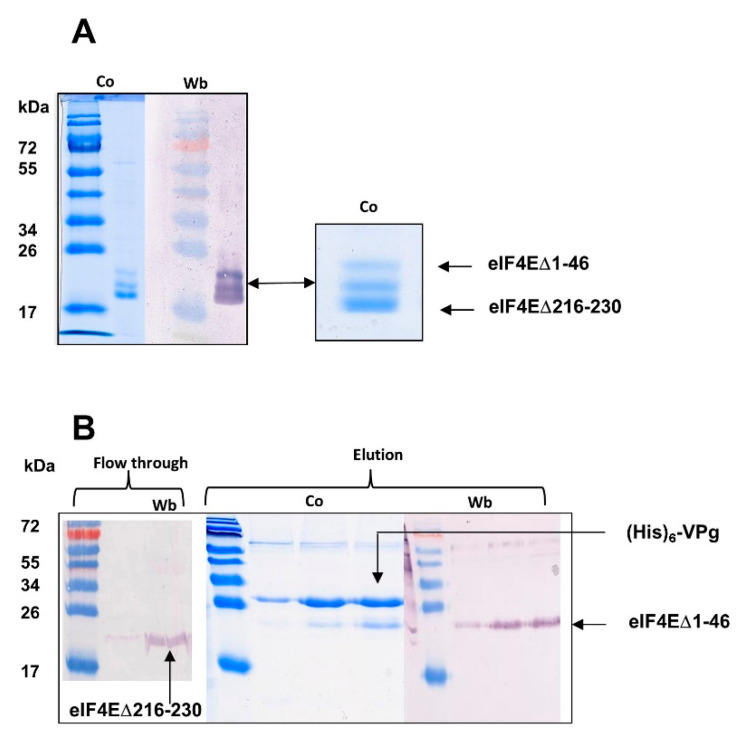
(**A**) Competition test of a mixture of eIF4E [∆1–46] and eIF4E[∆1–46/∆216–230] for m7GTP cap analog (**A**) and VPg (**B**) binding. (**A**) A mixture of both species was loaded on an m7GTP affinity column. The fraction eluted in 0.5 M KCl was analyzed by SDS-Page and Western blot. (**B**) A mixture of both species was incubated with (His)_6_-VPg and a Ni^2+^ IMAC was performed. The eIF4E [∆1–46/∆216–230] species was not retained (flow through). The eIF4E [∆1–46] species co-eluted with (His)6-VPg. Elution (250 mM imidazole). Fractions were analyzed by SDS-Page (Co) and Western blot Wt). Western blot revelation: lettuce eIF4E polyclonal antibodies. Subsequent analysis confirmed the identity of the two eIF4E species. The bands were cut from the gel and analyzed by mass spectrometry. Alternatively, they were transferred on a PVDF membrane and submitted to *N*-terminal sequencing. Both forms displayed the same *N*-terminal amino acid sequence.

A lettuce eIF4E *N*-terminus-deleted form, eIF4E [∆1–46]), binds to VPg with a similar affinity to the whole eIF4E, which precludes the eIF4E *N*-terminus involvement in VPg binding (Table 1). When a mixture of lettuce eIF4E [∆1–46] and of its C-ter truncated form eIF4E [∆1–46/∆216–230] was applied to a m7GTP agarose column, they were both retained, which confirmed the integrity of their cap-binding site. Both forms were also incubated with the LMV His_6_ VPg and affinity chromatographed on a Ni-NTA resin. The truncated eIF4E [∆1–46/∆216–230] was not retained and eluted in the flowthrough. The His_6_ VPg–eIF4E [∆1–46] complex was recovered after elution with imidazole (Figure 1).

**Figure 2:**
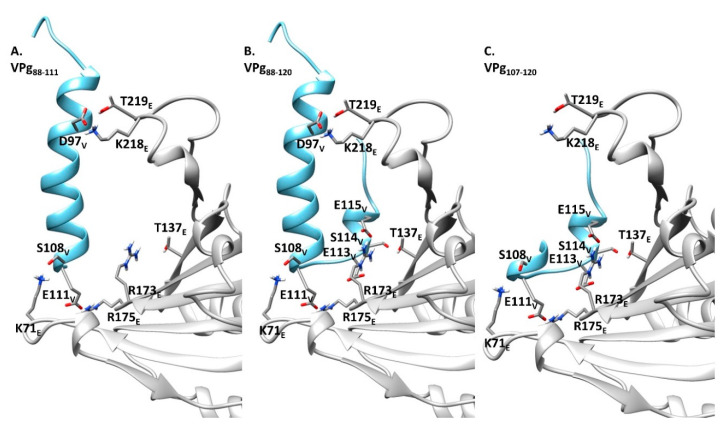
Models of interaction between the LMV VPg central domain (blue) and lettuce eIF4E (mo1^0^ isoform). (**A**–**C**), eIF4E interaction with VPg_88–111_, VPg_88–120_ and VPg_107–120_, respectively. The coordinates of the LMV VPg (LMV-AF199 isolate GenBank: AJ278854.1) and lettuce eIF4E (mo1^0^ isoform, GenBank: AF530162.1) were first derived by homology modeling from the VPg (PDB 6NFW) and eIF4E (*pisum sativum*, PDB 2WMC), respectively. The binary Complex was modeled by restraint-driven docking (Haddock web server), using a set of amino acids homologous to the ones identified elsewhere [21]. V and E subscripts refer to the residues belonging to VPg and eIF4E, respectively.

From this experiment, we deduced that the 15 last C-terminal amino acids of lettuce eIF4E are involved in the VPg binding. This observation confirms with a biologically relevant homologous complex (LMV VPg–lettuce eIF4E) what was earlier reported for the turnip mosaic virus VPg–*Caenorhabditis elegans* eIF4E heterologous complex [24]. During the preparation of this manuscript, the structure of VPg was solved by NMR spectroscopy, and the regions of VPg and eIF4E which overlap in the binary complex were mapped by 1H-15N HSQC bidimensional analysis [21]. The structural data confirmed that the region which interacts with VPg is on the opposite side of eIF4E *N*-ter domain. Using both the PDB coordinates of the newly solved structure of VPg from PVY (6nfw), and of human eIF4 (2gpq), we modeled the VPg from LMV-AF199 and eIF4E from lettuce. A docking of both proteins was attempted, using amino acids corresponding to the ones previously identified as being involved in the overlapping surface between VPg and eIF4E [21]. In the complex thus modeled, the positively charged residue K218 and T219 in eIF4E are facing D97 of VPg (Figure 2). The absence of these residues in eIF4E [∆1–46/∆216–230] could explain its defect in forming the complex with VPg. It emphasizes the importance of this interaction within the VPg helix to obtain a productive binary complex.

#### 2.1.2. Sites, Which in the VPg Central Region Determine Host Resistance Breakdown, Contribute to the Mechanism of VPg-eIF4E Interaction

Several attempts have been made to map the VPg regions involved in the interaction with eIF4E, either using yeast two-hybrid test [9], far western [25], fluorescence-based interaction measurements between eIF4E and deleted recombinant forms of VPg [26], ELISA interaction tests [18] or molecular modeling [27]. Although there are discrepancies, most of these studies point out the VPg central region as being part of the VPg–eIF4E interaction domain (Figure S2).

The model returned from NMR data provided strong elements of evidence that the VPg region involved in the interaction with eIF4E at least encompasses E98 to L118 in PVY VPg. By homology modeling and after energy minimization, this region corresponds from D97 to E115 in LMV VPg, as shown in Figure 1. In order to approach the underlying mechanism behind the interaction of the central part of LMV VPg with eIF4E, we compared the affinities of VPg_88–111_, VPg_88–120_ and VPg_107–120_ peptides for the lettuce eIF4E (Table 2). While the first two peptides display comparable affinities, VPg_107–120_, which bears the determinants of the resistance breakdown, has a 4-fold lower affinity for eIF4E. According to the model, the interaction lock between this fragment and eIF4E is mainly provided by two salt bridges formed between R173_E_ on the eIF4E side and E113_V_ and E115_V_ on the VPg side (Figure 2).

### 2.2. Kinetic Analysis of the VPg Central Domain Association with eIF4E

**Figure 3:**
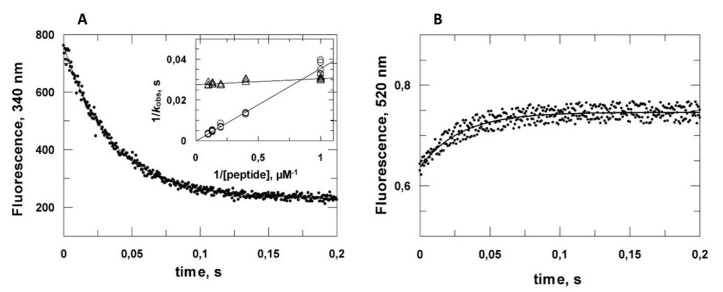
Stopped-flow traces of VPg central region association with eIF4E. (**A**) The quenching of eIF4E tryptophan fluorescence was monitored after mixing 0.3 µM eIF4E with 1 µM VPg_88–111_ (final concentration). The excitation wavelength was 283 nm. Inset, plots of 1/*k*_obs_ versus 1/[peptide] at 2.5, 5, 7.5 and 10 µM peptide concentration. VPg_88–111_ open circles, VPg_88–120_, open triangles. (**B**) Stopped-flow traces of VPg_88–111_*–eIF4E complex formation acquired at 520 nm upon excitation at 480 nm. VPg_88–111_*: doubly labeled fluorescent derivative of VPg_88–111_.

As seen above, VPg displays the same affinity for all eIF4E forms. The determinants of resistance breaking are located within the central domain of VPg. We hypothesized that the 107–120 region could modulate the mechanism of association, the details of which cannot be grasped through equilibrium thermodynamics. Consequently, fast kinetics measurement were performed by stopped flow to compare VPg_88–111_ and VPg_88–120_ association with eIF4E (Figure 3).

The eIF4E tryptophan fluorescence decrease concomitant to association was monitored for several peptide concentrations (Figure S3). A single exponential model fitted satisfactorily with the experimental data allowing the determination of *k*_obs_, the apparent constant of the complex formation. In the case of VPg_88–111_ association, a plot of 1/*k*_obs_ versus 1/[VPg_88–111_] gave a straight line, featuring the pseudo first order nature of the association depending on ligand concentration. By contrast, there was no significant dependence of *k*_obs_ upon VPg_88–120_ concentration, (Figure 3A inset). According to these data, the complex formation between VPg_88–111_ and eIF4E can be schematized as a one-step process:

(1) $$\mathrm{eIF}4E+{\mathrm{VPg}}_{88–111}\overset{k_{1}}{\to }\left[\mathrm{eIF}4E-{\mathrm{VPg}}_{88–111}\right]$$

with

(2) $$k_{\mathrm{obs}}=k_{1}\left[\mathrm{VPg}88-111\right]$$

while VPg_88–120_–eIF4E complex formation is compatible with the following scheme:

(3) $$\mathrm{eIF}4E+{\mathrm{VPg}}_{88–120}\overset{K_{D}}{⇌}\left[\mathrm{eIF}4E-{\mathrm{VPg}}_{88–120}\right]\;\left[\mathrm{eIF}4E-{\mathrm{VPg}}_{88–120}\right]\overset{k_{2}}{\to }\mathrm{eIF}4E-{\mathrm{VPg}}_{88–120}$$

(4) $$k_{\mathrm{obs}}=k_{2}\frac{\left[{\mathrm{VPg}}_{88–120}\right]}{K_{D}+\left[{\mathrm{VPg}}_{88–120}\right]}$$

### 2.3. Reassessment of the Binding Properties of the VPg Central Region with eIF4E

**Figure 4:**
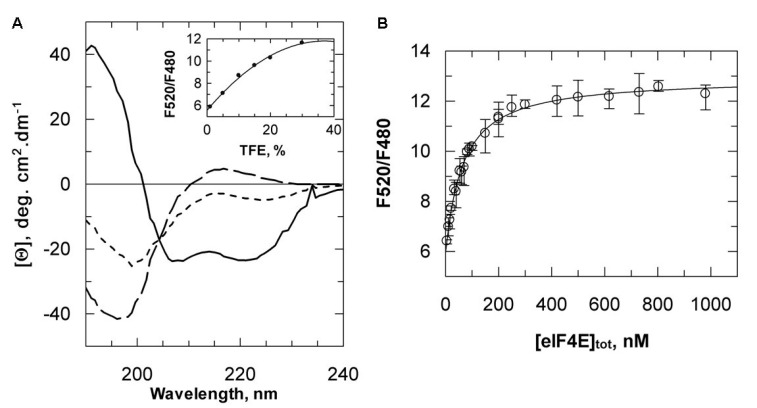
VPg_88–111_ propensity to fold into an α-helical conformation. (**A**) Far UV-CD spectra of VPg_88–111_* in the absence (dashed line), or in the presence of 10% (dotted line) and 20% (solid line) of TFE. Inset, AEDANS to fluorescein RET increase upon TFE addition (mean of three to six measurements). (**B**) Steady state titration of eIF4E binding to VPg_88–111_*. The solution contained 250 nM of VPg_88–111_*. AEDANS was excited at 337 nm. The solid line represents the best fit of the simple bimolecular equilibrium model to the experimental data.

We previously reported that the LMV VPg central region interacts specifically with lettuce eIF4E. The structure of this domain was predicted to include an amphipathic α-helix [18], which was confirmed in the VPg solved structure [21]. In the LMV VPg, this helix corresponds to VPg_88–111_. This small polypeptide segment is predicted to be on the boundary between an ordered region and the disordered central region within the VPg [28]. Because of entropy prevalence, short monomeric peptides less than 20 residues in length are unlikely to spontaneously adopt an α-helical conformation in aqueous solution. We hypothesized that the association with eIF4E could provide the thermodynamic driving force required for the folding. We report here an attempt to probe the possible folding of VPg_88–111_ into an α-helix upon association by resonance energy transfer (RET). It was anticipated that, if an energy donor (D) and its acceptor (A) were positioned respectively at the N and C terminus ends of the peptide, D and A could be closer upon a random coil to helix transition. This transition could, in principle, be associated with a RET increase between D and A. Preliminary calculations using several 20 amino acid random coil conformer ensembles available from the PDB returned an average distance (*r*) of 38.4 Å between the 20 mer N and C terminus ends. Upon folding into α-helix, *r* decreases to about 25 Å. The choice of the D–A pair is determined by the Forster distance *R*_0_ at which D fluorescence emission would be decreased half its intensity. Because the energy transfer *E* depends strongly on the distance *r* between D and A, RET can only be used to measure *r* if it is in the range of 0.5 *R*_0_ to 2*R*_0_ [29]. *R*_0_ values of many tryptophan-A pairs are close to the expected Nter-Cter distance. However, as eIF4E contains nine tryptophan residues, a D–A couple which gives signal out of their intrinsic fluorescence properties was necessary. AEDANS and 5-acetamido-fluorescein (5-AF) were chosen as D and A, respectively (*R*_0_ 46–54 Å) [30]. A modified form of VPg_88–111_ was designed with two cysteine residues, one at the peptide *N*-terminal end and one after glutamate 111 for D and A coupling (CSVFSDIGLVQDAFGKERLKLLSGGECY). A tyrosine residue was added for convenient quantitation. Formation of α-helices in disordered polypeptides results from a complex sequence of energetically unfavorable initial events [31]. Indeed, the dichroic spectrum of the doubly labeled VPg_88–111_ derivative (VPg_88–111_*) results from an equilibrium between random coil conformers. The secondary structure stabilizer 2,2,2-trifluoroethanol (TFE) was used to test the propensity of VPg_88–111_* to fold into an α-helical conformation. In 20% TFE, up to 70% of the spectrum was characteristic of an α-helix (Figure 4A). Importantly, a RET signal was observed proportional to the increase in TFE concentration, validating that the random coil to α-helix transition was associated with an increase in the energy transfer efficiency between AEDANS and fluorescein (Figure 4A inset). It was not possible to use circular dichroism to titrate the binding of eIF4E as the large eIF4E dichroic signal masked the folding of VPg_88–111_*. However, an increase in the RET signal proportional to the amount of eIF4E added was observed, and a steady state titration of eIF4E binding to VPg_88–111_* could readily be obtained (Figure 4B). A *K*_D_ value of 63 ± 3 nM was determined, in good agreement with values measured for the whole VPg from LMV-AF199 strain (Figure S4).

**Figure 5:**
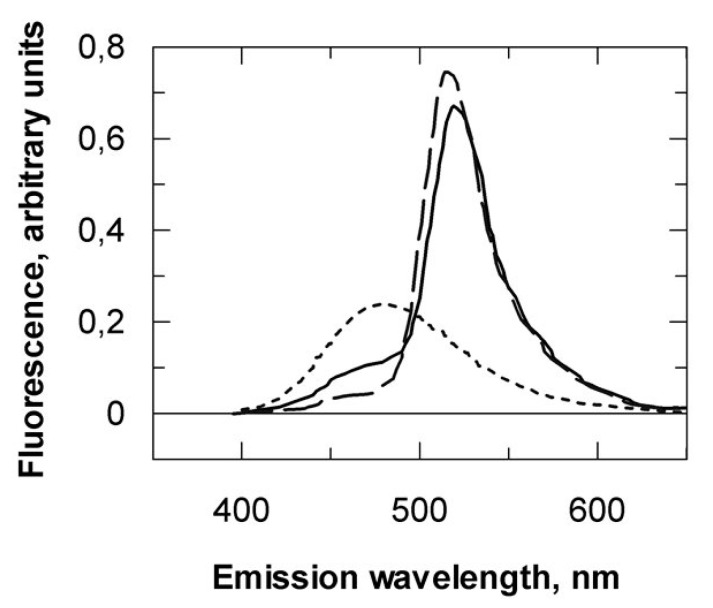
Fluorescence characteristics of labeled VPg_88–111_. Emission spectra of 0.5 µM monolabeled AEDANS-VPg_88–111_ (dotted line), and dual-labeled VPg_88–111_* either alone (solid line) or after mixing with 1 µM eIF4E (dashed line). Excitation was 340 nm.

A large RET signal was observed for VPg_88–111_* random coil state with a transfer efficiency *E_c_* of 65%. An AEDANS-fluorescein *R*_0_ value of 42 Å was determined. A *r*_c_ value of 38.5 ± 1.5 Å was obtained for the AEDANS-fluorescein distance in the VPg_88–111_* random coil state. When VPg_88–111_* was mixed with a 2-fold molar excess of eIF4E, the transfer efficiency *E_h_* reached 93% and a *r*_h_ value of 27 ± 2.1 Å was obtained between the two probes within the VPg_88–111_*–eIF4E complex. The emission spectra showed a 4 nm blue shift in the VPg_88–111_*–eIF4E complex. The probes could be in a less-polar environment in the complex, possibly due to an overlap between the hydrophobic side of the helix and the eIF4E surface (Figure 5).

RET associated with the transition was also monitored by stopped flow, giving an apparent rate constant of comparable value to the one determined by measuring eIF4E intrinsic fluorescence decrease accompanying its binding with unlabeled VPg_88–111_ (Figure 3B).

Our results show that the VPg_88–111_ helix is part of the overlap between VPg and eIF4E. According to our structural model (Figure 1), it is likely that the driving force enabling VPg_88–111_ helix folding upon binding results from the contribution of three major amino acid clusters, namely (D97_V_, K218_E_, T219_E_), (S108_V_, K71_E_) and (E111_V_,R175_E_). It must be stressed that this helix pre-exists in the monomeric VPg, as shown by the NMR data [21].

Considering the two-step mechanism, and according to Equation (4), one can see that, if the first association step is very fast (*K*_D_ on the nmolar order), then *k*_obs_ is close to *k*_2_, the rate constant, of the VPg_88–120_ conformational transition between a random coil and a conformation that, according to our data, is compatible with an α-helix. The value of *k*_2_ was close to 33 s^−1^, which is at least one order of magnitude slower than what is usually reported for the helical folding of a 20 amino acid peptide in solution [32]. Helix formation was depicted as the sum of a complex interplay between a few different types of propagation of short nucleation events along the chain with energetically unfavorable formation of a discreet number of turns being rate limiting [31]. However, in contrast to most of the studies performed with peptides free in solution with no external steric constraints, in the model discussed here, the folding of VPg_88–111_* is driven by its interaction with eIF4E. Consequently, with respect to VPg_88–120_, the fast first association step evidenced here could immobilize a recognition motif present in the 107–120 part of VPg_88–120_ on the surface of eIF4E. This decrease in the degree of freedom of the chain could impact significantly the subsequent folding kinetics. The intrinsically disordered C-terminal domain of the measles virus nucleoprotein (NTAIL) folds into a helical conformation upon binding with the X domain (XD) of the viral phosphoprotein [33]. The two-step binding mechanism is very similar to the one investigated here. The first association step involves microsecond processes that are shorter than what the stopped flow technics can measure. Using a T jump procedure, it was possible to reveal a hyperbolic ligand dependency of the pseudo first-order rate constant and a value of 1 ms was determined for the terminal folding step that is comparable in magnitude to the value of 30 ms we obtained (1/*k*_2_) [34].

Although in the whole VPg molecule, the folding upon binding of helix 88–111 is unlikely, the preliminary 107–120 segment docking could drive the positioning of this “preformed helix”. As highlighted in the LMV lettuce pathosystem, host resistance is not systematically associated with an impairment of the VPg–eIF4E complex formation, but rather to a modulation of this interaction. Amino acid substitutions within the intrinsically disordered VPg_107–120_ region could tune virus adaptation to its host. A co-evolution was previously reported between this region and eIF4E. Therefore, the virus resistance-breakdown determinants located in the 107–120 segment of VPg_88–120_ could be part of the specific recognition motive involved in the first kinetic step of the VPg–eIF4E association.

## 3. Experimental Procedures

### 3.1. Protein Preparation

The eIF4E initiation factor from lettuce (*Trocadero cultivar*) and its derived molecular species were cloned into the vector pENTR/D-TOPO^®^ (Invitrogen, Carlsbad, CA, USA) [35]. They were transferred into pDEST^TM^17 using the Gateway^®^ recombinant technology to allow production of *N*-terminal fusions with a hexahistidine tag (Invitrogen). In addition, full-length eIF4E and eIF4E∆(1–46) were cloned into the vector pENTR/SD/D-TOPO^®^ (Invitrogen) and transferred into the Gateway pDEST^TM^14 expression vector according to the manufacturer’s instructions to obtain untagged full-length eIF4E and eIF4E∆(1–46). The constructs were introduced into *E. coli* (BL21-AI strain), and expression and purification of the His-tagged proteins were performed on ion metal affinity chromatography followed by m7GTP-Sepharose 4BGE Healthcare (Chicago, IL, USA) as previously described [36]. Untagged proteins were obtained by one-step affinity purification on m7GTP sepharose 4B. The lettuce mosaic virus VPg (LMV-AF199 strain) translated region was cloned into the pTrcHis C expression vector downstream from a hexahistidine tag (Invitrogen). The vector contains a specific anterokinase cleavage site in frame with the protein for proteolytic tag removal. The protein was produced and purified as previously reported [36] except that the final monoQ chromatographic step was replaced by a size exclusion chromatography on a Superdex 75 HR 10/30 column (GE Healthcare, Chicago, IL, USA) in 20 mM Hepes pH 8, 300 mM NaCl and 2 mM DTT. For His-tag removal, this last step was omitted and the protein was diluted twice in the same buffer with reduced ionic strength (150 mM NaCl) containing 1 mM CaCl2, 0.1% Tween-20. His-tagged enterokinase (0.02 mg for 0.2 mg VPg) was added and the protein mix was dialyzed overnight at 4 °C against the same buffer. The protease and uncleaved tagged VPg were subsequently trapped on a NiNTA resine and the pure free VPg form was recovered in the flow through (30–40% yield).

### 3.2. VPg_(88–111)_ Dual Labeling

The VPg_(88–111)_ derivative, carrying N and C terminal cysteine residues (CSVFSDIGLVQDAFGKERLKLLSGGECY) was sequentially labeled with IAEDANS and 5-iodoacetamido-fluorescein (5-IAF) as follows. A 3-fold molar excess of IAEDANS was first reacted with the peptide as described above. The peptides were separated from unreacted IAEDANS and small byproducts by desalting on a G25 column. Singly labeled peptide was separated from unlabeled and doubly labeled peptide on a C18 LiChroCART 250-4 Superspher (0.4 × 25 cm, porosity of 100 Å) column (Merck, Darmstadt, Germany). The column was equilibrated in 80% medium A containing 0.06% TFA in water and 20% medium B containing 50% acetonitrile and 0.04% TFA in water. The sample was injected onto the column, and after a 5 min wash, a 20 to 50% B gradient was applied over 15 min, at a flow rate of 1 mL/min. An average of 25% of the starting peptide was recovered as pure singly labeled species (as confirmed by electrospray ionization mass Spectrometry). This fraction was concentrated by freeze drying, dissolved in 25 mM HEPES, pH 7.5 and labeled by addition of a 5 molar excess of 5-IAF using the same procedure as for IAEDANS labeling. The doubly labeled peptide was purified by HPLC (40% yield) as above and stored after freeze drying under N_2_ at −20 °C in the dark until use.

### 3.3. Circular Dichroism

Data were recorded on a Jasco J-815 dichrograph in 10 mM phosphate buffer pH 7.5, 100 mM NaF at 25 °C. The peptide concentration was 0.1 mg/mL and the cuvette path length was 1 mm. Data interval 0.5 nm, bandwith 1 nm, and scanning speed 50 nm/min. Each spectrum was the average of 10 scans. Data were processed using the online server Dichroweb, (http://dichroweb.cryst.bbk.ac.uk/html/links.shtml) [37].

### 3.4. Fluorescence Measurements

All steady state fluorescence acquisitions were obtained at 25 °C in 20 mM HEPES pH 7.5, 0.25 mM NaCl and 1 mM DTT using a SAFAS Xenius spectrofluorimeter (Monaco) equipped with a Peltier temperature controller. For optical characteristics of the instrument, see [36]. Affinity constants were deduced from steady state eIF4E tryptophan intrinsic fluorescence decrease upon titration by VPg as previously described [36]. Fast kinetics of VPg_88–106_ and VPg_88–120_ association to eIF4E were monitored on a MOS-200 spectrometer equipped with a SFM 3000 stopped-flow device, dead time 2 ms (BioLogic, Grenoble, France). 

### 3.5. Fret Measurements

Energy transfer efficiency. Energy transfer was determined from both increase in acceptor fluorescence and decrease in donor fluorescence in order to ensure that all the changes observed were due to energy transfer and were not environmental or quenching effects [38]. Two methods were thus used to determine the resonance energy transfer efficiency (*E*) between the AEDANS and AF probes. In the first method, the AEDANS (donor) was excited at 337 nm and fluorescence intensity at 480 nm was measured in the absence (*F*_D_) and then in the presence (*F*_DA_) of the acceptor (AF). Any reduction in the latter was assumed to be due to energy transfer to AF. Since the labeling efficiency is generally not 100%, *E* is calculated from the following relationship [39]:

(5) $$E=\left(1-\frac{F_{\mathrm{DA}}}{F_{D}}\right)\;\frac{1}{f_{A}}$$

where *f_A_* is the fractional occupancy of the acceptor site which was set to 1 as the dual-labeled peptide was assumed to be pure (see above). In the second method, the increase at 493 nm in the AF excitation spectrum caused by transfer from AEDANS was used to calculate *E* according to the following relationship [38]:

(6) $$E=\left(\frac{F_{337}}{F_{493}}-\frac{\varepsilon_{A337}}{\varepsilon_{A493}}\right)\;\frac{\varepsilon_{A493}}{\varepsilon_{D337}}\;$$

where *F*_337_ and *F*_493_ are the corrected intensities of the excitation spectrum, and ε_A337_ (9.6 × 10^3^ M^−1^ cm^−1^) and ε_A493_ (77 × 10^3^ M^−1^ cm^−1^) are the AF extinction coefficients, at 337 nm (AEDANS excitation wavelength) and 493 nm (AF excitation wavelength), respectively. ε_D337_ (5.7 × 10^3^ M^−1^ cm^−1^) is the extinction coefficient of the donor AEDANS at 337 nm.

Calculation of *r*, the donor–acceptor distance. The D–A distance, *r*, can be extracted from the following relationship after experimental determination of *R*_0_:

(7) $$E=\frac{R_{0}^{6}}{R_{0}^{6}+r^{6}}$$

where *R*_0_ is the Förster critical distance at which *E* is 50%. The transfer efficiency is strongly dependent on distance when the D–A distance is near *R*_0_.

The Förster distance is given by:

(8) $$R_{0}=0.211{\left(\kappa^{2}n^{-4}Q_{D}J\left(\lambda \right)\right)}^{1/6}$$

with *n* being the refractive index for biomolecules in aqueous solution assumed to be 1.4 and *κ*^2^ the orientation factor. The term *κ*^2^ is a factor describing the relative orientation in space of the transition dipoles of the donor and acceptor. Segmental motions of D and A probes bound to macromolecules tend to randomize the orientations. The term *A*_0_ is used to refer to the anisotropy observed in the absence of other depolarizing processes such as rotational diffusion or energy transfer. Anisotropy measurements were used to estimate *A*_0_ values from two Perrin’s plots of AEDANS or AF singly labeled VPg_88–106_ upon excitation at 337 and 493 nm, respectively. These two *A*_0_ values were in between 0.25 and 0.31. For fluorophores with mixed polarization, where *A*_0_ < 0.3, the error in distance is thought to be below 10%. Therefore, we assumed that D and A probes bound at the *N*-terminal and C-terminal ends of VPg_88–106_ randomize by rotational diffusion prior to energy transfer. In such a case, the value of *κ*^2^, the orientation factor is generally set to 2/3.

Estimation of *Q*_D_, AEDANS (D) quantum efficiency. Emission spectra of known concentrations of VPg_88–106_ coupled to AEDANS only (no acceptor) and of quinine bisulfate taken as reference were monitored upon excitation at 337 nm. The quantum efficiency of AEDANS linked to VPg_88–106_ was calculated as follows:

(9) $$Q_{D}=\frac{Ar_{D}}{Ar_{Q}}\frac{0.7A_{Q}^{337}}{A_{\mathrm{VPg}}^{337}}$$

with *Ar*_D_ and *Ar*_Q_ the areas under the emission spectra of AEDANs-VPg_88–106_ and quinine bisulfate, respectively. $A_{Q}^{337}$ and $A_{\mathrm{VPg}}^{337}$ are the absorbance values of AEDANS-VPg_88–106_ and quinine bisulfate, respectively [40]. *J*(*λ*), the spectra overlap integral (in cm^3^ M^−1^), is given by:

(10) $$J\left(\lambda \right)=\frac{\int_{0}^{\infty }F_{D}\left(\lambda \right)\varepsilon_{A}\left(\lambda \right)\lambda^{4}d\lambda }{\int_{0}^{\infty }F_{D}\left(\lambda \right)d\lambda }$$

where *F*_D_(*λ*) is the fluorescence of VPg_88–106_ dual labeled excited at 337 nm and ε_A_ is the extinction coefficient of the attached AF expressed in M^−1^ cm^−1^. *J* was numerically integrated at 3 nm intervals.

### 3.6. Data Analysis

Interaction and kinetic models were examined by fitting the associated equations to experimental data using non-linear regression using the Graphit package (Erithacus Ltd., Horley, UK). Affinity and kinetic constants were determined from the model giving the best fit to the data.

### 3.7. Protein Modeling

The binary complex between the LMV VPg and lettuce eIF4E was built as follows: the coordinates of the LMV VPg (LMV-AF199 isolate GenBank: AJ278854.1) and lettuce eIF4E (mo1^0^ isoform, GenBank: AF530162.1, *cultivar Salinas*) were first derived by homology modeling, starting from the PVY VPg (PDB 6NFW) and eIF4E (*pisum sativum*, PDB 2WMC, best score), respectively, using Phyre 2; http://www.sbg.bio.ic.ac.uk/phyre2 [41]. Model repair and energy minimization of both proteins were performed using the Foldx module included in the standalone Yasara pack [42]; http://www.yasara.org.

**Figure 6:**
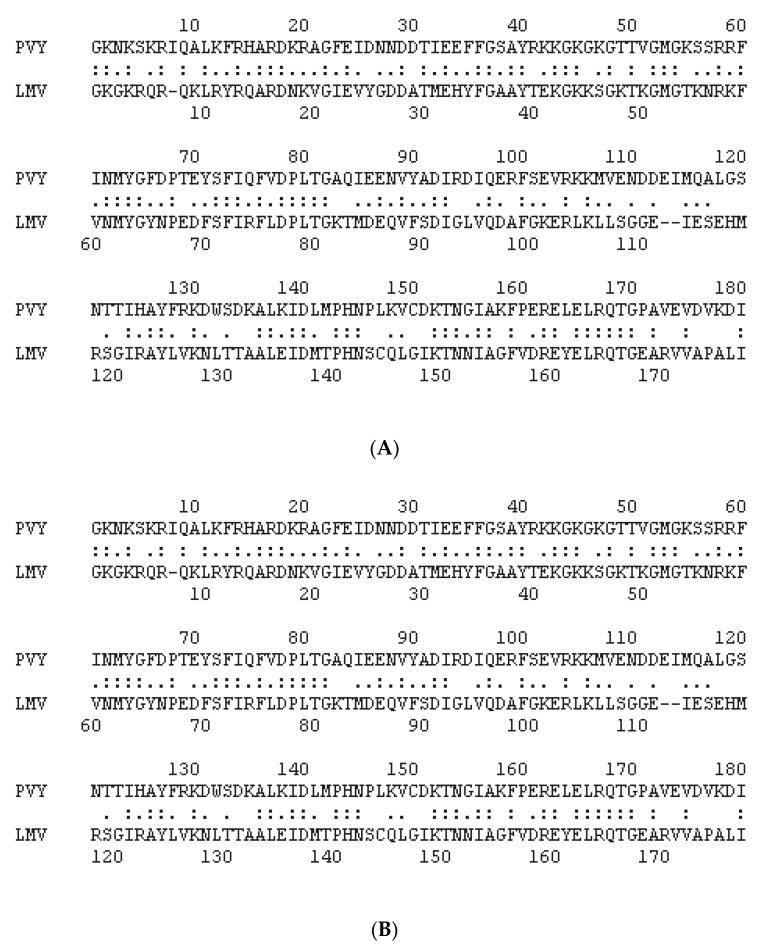
(**A**) Alignment between VPg from PVY, N strain, UniProt, P18247, used to solve the VPg structure and VPg from LMV-AF199, E strain, UniProt P89876, used to build the homology model in this study. (**B**) Alignment between eIF4E, *Homo sapiens*, UniProt P06730 and lettuce eIF4E (Salinas cultivar) UniProt Q7XJB1_LACSA.

An examination of the PVY VPg–human eIF4E model [21] helped to identify amino acids directly involved in the interaction through salt bridges and hydrogen bonds. Structural alignments between human and eIF4E lettuce, on the one hand, and between PVY VPg and LMV VPg, on the other hand, allowed the identification in eIF4E lettuce and LMV VPg of residues homologous to those involved in the human eIF4E–PVY VPg binary complex (Figure 6 and Table 3).

These residues were used for modeling the binary complex by restraint-driven docking; http://milou.science.uu.nl/services/HADDOCK2.2/ [43]. The geometry of the interacting residues’ side chains and that of their surrounding residues within a radius of 5 Å were further optimized by energy minimization using the “structure editing” module of Chimera (standalone version v1.14); http://www.rbvi.ucsf.edu/chimera [44].

## Figures and Tables

**Table 1:** Thermodynamic constants of dissociation between the LMV VPg from the non-resistance-breaking strain (LMV0) and various forms of lettuce eIF4E.

Dissociation Constants (*K*_D_ × 10^9^, mol/L) at 25 °C		
	VPg	m7GTP
eIF4E^0^	242 ± 70	307 ± 27
eIF4E^1^	340 ± 80	261 ± 43
eIF4E^2^	288 ± 30	351 ± 18
eIF4E^0^ [∆1–46]	276 ± 17	242 ± 33
eIF4E^0^-m7GTP ^♦^	746 ± 98	nd
eIF4E^0^–VPg ^♦^	nd	3000 ± 239
eIF4E^1^-m7GTP ^♦^	632 ± 29	nd
eIF4E^2^-m7GTP ^♦^	703 ± 55	nd

^♦^ eIF4E 0.5 µM was pre-incubated with either m7GTP or VPg 2 µM prior to titration. “nd”: not determined.

**Table 2:** Synthetic peptides corresponding to the VPg region involved in the VPg-eIF4E interaction.

	Sequence	*K*_D_ (nmol/L)
VPg_88–111_	VFSDIGLVQ**D**AFGKERLKLL**S**GG**E**	72 ± 11
VPg_88–120_	VFSDIGLVQ**D**AFGKERLKLL**S**GG**E**I**ESE**HMRSG	67 ± 7
VPg_107–120_	L**S**GG**E**I**ESE**HMRSG	852 ± 57

The synthetic peptides 88–111, 88–120 and 107–120 affinities (*K*a) for eIF4E were deduced from monitoring the protein intrinsic fluorescence decay upon binding (see material and methods). These peptides were derived from the VPg sequence, LMV strain AF199. The residues directly involved in the interaction with eIF4E are in bold.

**Table 3:** Interacting residues in the PVY VPg–human eIF4E complex, and their counterpart used to dock the LMV VPg–lettuce eIF4E complex.

PVY VPg–Human eIF4E		LMV VPg–Lettuce eIF4E	
eIF4E	VPg	VPg	eIF4E
K206	E98	D97	K218
S207	E98	D97	T219
K52	E109	S108	K71
K159	D111	E111	R175
R157	E114	E113	R173
K159	E114	E113	R175
R157	L118	E115	R173
R157 T116	M115 M115	S114 S114	R173 T137

## Supplementary Materials

The following are available online at https://www.mdpi.com/1422-0067/21/16/5618/s1, Figure S1: Comparison of all interaction combinations between eIF4E from resistant and susceptible lettuces and VPgs from resistance or non-resistance breaking LMVs; Figure S2: Mapping of eIF4E region involved in the interaction with VPg; Figure S3: Transient kinetics of binary complex formation between eIF4E (0.3 µM) and VPg88–111 or VPg88–120; Figure S4: Steady state titration of lettuce eIF4E association with VPg from LMV.

## Abbreviations

LMV	lettuce mosaic virus
PVY	potato virus Y
TEV	tobacco etch virus
TuMV	turnip mosaic virus
IAEDANS	*N*′-(iodoacetyl)-*N*′-(5-sulfo-1-naphtyl)ethylenediamine
His_6_ eIF4E	*N*-ter hexahistidine-tagged lettuce eIF4E
His_6_ VPg	*N*-ter hexahistidine-tagged LMV VPg
eIF4E^Δ1−46^	untagged lettuce eIF4E deleted from its first 46 *N*-ter amino acids
His_6_ VPg*	IAEDANS-labeled *N*-ter hexahistidine-tagged LMV VPg
His_6_ eIF4E*	IAEDANS-labeled *N*-ter hexahistidine-tagged lettuce eIF4E
eIF4E*	IAEDANS-labeled untagged lettuce eIF4E
eIF4E^Δ1−46^*	IAEDANS-labeled untagged lettuce eIF4E deleted from its first 46 *N*-ter amino acids
[His_6_ VPg*–His_6_ eIF4E]	IAEDANS-labeled binary complex
