# Supplementary material for: Spectroscopic Investigation of the Kinetic Mechanism Involved in the Association of Potyviral VPg with the Host Plant Translation Initiation Factor eIF4E
Source: Int J Mol Sci. 2020 Aug 5;21(16):5618. doi: 10.3390/ijms21165618 (PMC7460627; doi:10.3390/ijms21165618)
Supplement: Supplementary file 1 [file ijms-21-05618-s001.pdf]

International Journal of *Molecular Sciences*

MDPI

Article

Spectroscopic Investigation of the Kinetic Mechanism Involved in the Association of Potyviral VPg with the Host Plant Translation Initiation Factor eIF4E

Jocelyne Walter [1], Amandine Barra [1], Justine Charon [2], Geneviève Tavert-Roudet [1] and Thierry Michon[1,*]

[1] University of Bordeaux, INRAE, Biologie du Fruit et Pathologie, UMR 1332, F–33140 Villenave d'Ornon, France

[2] University of Sydney, Faculty of Sciences, Charles Perkins Center D17, Camperdown campus, 2026 NSW, Australia

Supporting Information

Figure S1: Comparison of all interaction combinations between eIF4E from resistant and susceptible lettuces and VPgs from resistance or non-resistance breaking LMVs.

Figure S2: Mapping of eIF4E region involved in the interaction with VPg.

Figure S3: Transient kinetics of binary complex formation between eIF4E (0.3 μM) and VPg88–111 or VPg88-120.

Figure S4: Steady state titration of lettuce eIF4E association with VPg from LMV.

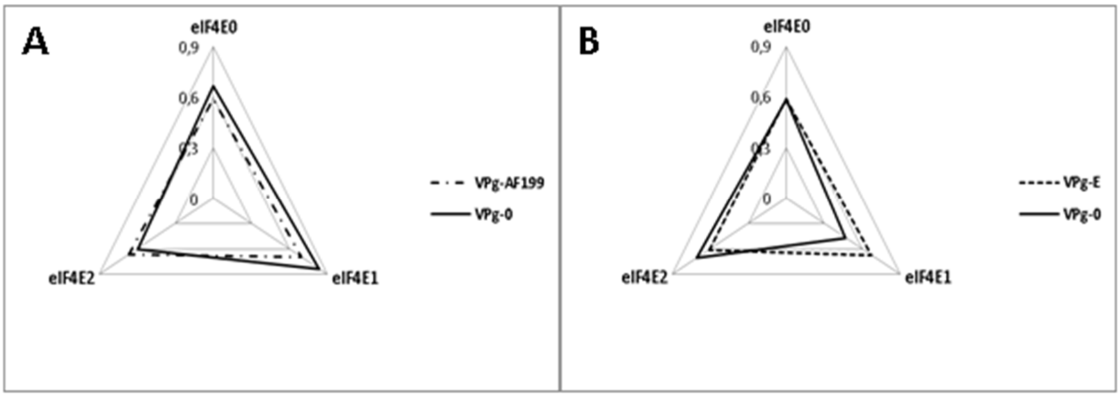

Figure S1. Comparison of the interactions between eIF4E from susceptible (eIF4^0) or resistant (eIF4E^1, eIF4E^2) lettuce cultivars and VPg from non-resistance-breaking (VPg0) or resistance-breaking LMV isolates (VPgAF199 and VPgE). Interactions were assessed by ELISA-based assays, as described previously [1]. (**A**)VPgAF199 versus VPg0. (**B**) VPgE versus VPg0. Purified eIF4Es bait (4 μg ml^{-1}) were immobilized in the wells, and VPgs prey (6μg ml^{-1}) were added. Interactions were revealed using a combination of a monoclonal antibody directed against LMV-VPg (mAb 1H5) and anti-mouse alkaline phosphatase conjugates. Values in the graphs correspond to absorbance at 405 nm after background noise subtraction (no immobilized bait protein in the wells). All proteins were prepared following the protocol detailed in the material and method section, except that the His tag was not removed.

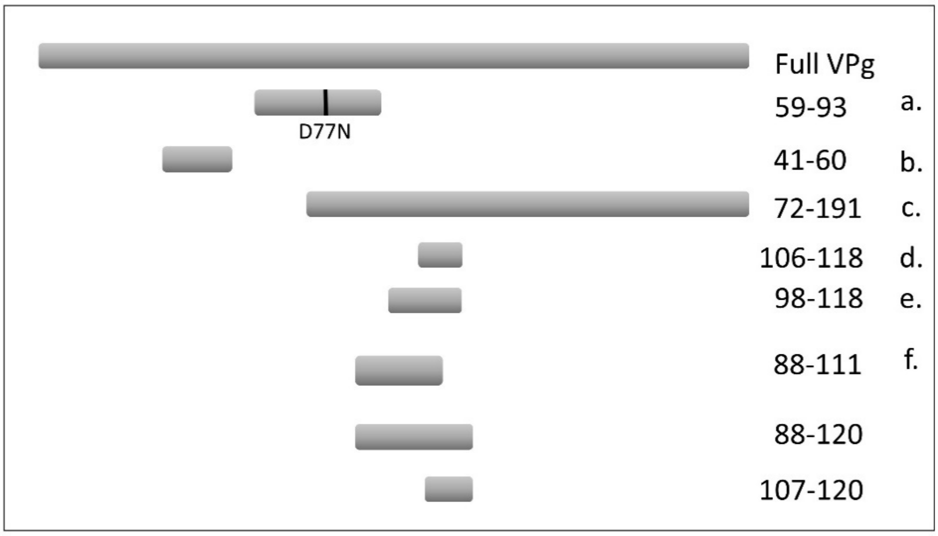

Figure S2. Scheme of the VPg regions proposed as being involved in the interaction with eIF4E. (**a**). TuMV, D77N mutation was reported to prevent TuMV infectivity [2]; (**b**). PVY [3]; (**c**). TuMV [4]; (**d**). TEV [5]. (**e**). PVY [6]. (**f**). Synthetic peptides derived from the LMV VPg central region, tested in this study for their ability to bind eIF4E from lettuce.

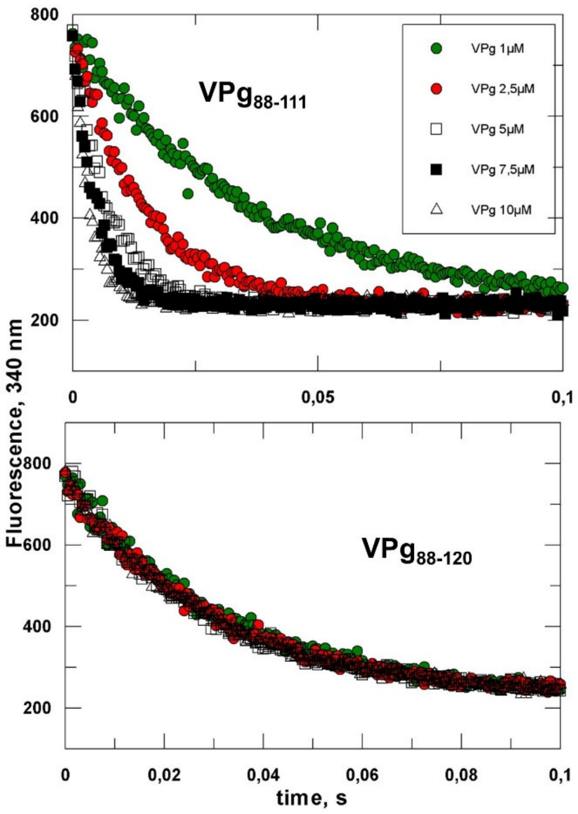

Figure S3. Transient kinetics of binary complex formation between eIF4E (0.3 µM) and VPg88–111 or VPg88–120. Peptide concentrations varied between 1 and 10 µM.

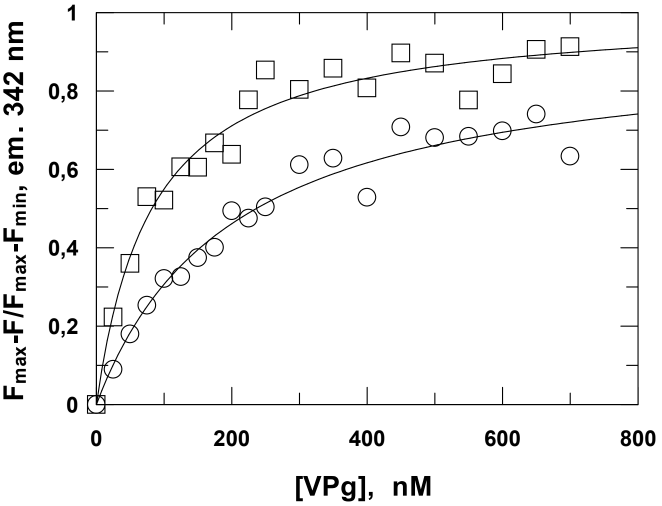

Figure S4. Steady state titration of lettuce eIF4E association with VPg from LMV. Open circle, VPg from non-resistance-breaking virus strain LMV0. Open square, VPg from resistance-breaking virus strain LMV AF199. Measurements were performed as in [7]. K_D was 202 ± 28 nM and 82 ± 10 nM for VPg of LMV0 and AF199 strains, respectively.

References

1. Roudet-Tavert, G.; Michon, T.; Walter, J.; Delaunay, T.; Redondo, E.; Le Gall, O. Central domain of a potyvirus VPg is involved in the interaction with the host translation initiation factor eIF4E and the viral protein HcPro. *J. Gen. Virol.* **2007**, *88*, 1029–1033.
2. Leonard, S.; Plante, D.; Wittmann, S.; Daigneault, N.; Fortin, M.G.; Laliberte, J.F. Complex formation between potyvirus VPg and translation eukaryotic initiation factor 4E correlates with virus infectivity. *J. Virol.* **2000**, *74*, 7730–7737.
3. Grzela, R.; Strokovska, L.; Andrieu, J.P.; Dublet, B.; Zagorski, W.; Chroboczek, J. Potyvirus terminal protein VPg, effector of host eukaryotic initiation factor eIF4E. *Biochimie* **2006**, *88*, 887–896, doi:10.1016/j.biochi.2006.02.012.
4. Khan, M.A.; Miyoshi, H.; Gallie, D.R.; Goss, D.J. Potyvirus genome-linked protein, VPg, directly affects wheat germ in vitro translation: Interactions with translation initiation factors eIF4F and eIFiso4F. *J. Biol Chem.* **2008**, *283*, 1340–1349, doi:10.1074/jbc.M703356200.
5. Perez, K.; Yeam, I.; Kang, B.C.; Ripoll, D.R.; Kim, J.; Murphy, J.F.; Jahn, M.M. Tobacco etch virus infectivity in Capsicum spp. is determined by a maximum of three amino acids in the viral virulence determinant VPg. *Mol. Plant.-Microbe Interact.* **2012**, *25*, 1562–1573, doi:10.1094/MPMI-04-12-0091-R.
6. Coutinho de Oliveira, L.; Volpon, L.; Rahardjo, A.K.; Osborne, M.J.; Culjkovic-Kraljacic, B.; Trahan, C.; Oeffinger, M.; Kwok, B.H.; Borden, K.L.B. Structural studies of the eIF4E–VPg complex reveal a direct competition for capped RNA: Implications for translation. *Proc. Natl Acad Sci.* **2019**, *116*, 24056–24065, doi:10.1073/pnas.1904752116.
7. Michon, T.; Estevez, Y.; Walter, J.; German-Retana, S.; Le Gall, O. The potyviral virus genome-linked protein VPg forms a ternary complex with the eukaryotic initiation factors eIF4E and eIF4G and reduces eIF4E affinity for a mRNA cap analogue. *FEBS* J. **2006**, *273*, 1312–1322.

© 2020 by the authors. Licensee MDPI, Basel, Switzerland. This article is an open access article distributed under the terms and conditions of the Creative Commons Attribution (CC BY) license (http://creativecommons.org/licenses/by/4.0/).
